# Enhanced coordination of care to reduce medication risks in older home care clients in primary care: a randomized controlled trial

**DOI:** 10.1186/s12877-019-1353-2

**Published:** 2019-11-27

**Authors:** Terhi Toivo, Marja Airaksinen, Maarit Dimitrow, Eeva Savela, Katariina Pelkonen, Valtteri Kiuru, Tuula Suominen, Mira Uunimäki, Sirkka-Liisa Kivelä, Saija Leikola, Juha Puustinen

**Affiliations:** 1 Faculty of Pharmacy, Division of Pharmacology and Pharmacotherapy, Clinical Pharmacy Group, University of Helsinki, Viikinkaari 5 E, P.O. BOX 56, 00014 Helsinki, Finland; 2 1st Pharmacy of Lohja, Laurinkatu 37-41 A, 08100 Turku, Finland; 3 City of Lohja, Services for Aged Residents, PL 71, 08101 Lohja, Finland; 4 Institute of Clinical Medicine, Department of Family Medicine, University of Turku, 20014 University of Turku, Finland; 5 Satakunta Hospital District, Satakunta Central Hospital, Unit of Neurology, Sairaalantie 3, 28500 Pori, Finland

**Keywords:** Coordination of care, Medication risk management, Home care, Older adults, Medication safety

## Abstract

**Background:**

As populations are aging, a growing number of home care clients are frail and use multiple, complex medications. Combined with the lack of coordination of care this may pose uncontrolled polypharmacy and potential patient safety risks. The aim of this study was to assess the impact of a care coordination intervention on medication risks identified in drug regimens of older home care clients over a one-year period.

**Methods:**

Two-arm, parallel, cluster randomized controlled trial with baseline and follow-up assessment at 12 months. The study was conducted in Primary Care in Lohja, Finland: all 5 home care units, the public healthcare center, and a private community pharmacy. Participants: All consented home care clients aged > 65 years, using at least one prescription medicine who were assessed at baseline and at 12 months. Intervention: Practical nurses were trained to make the preliminary medication risk assessment during home visits and report findings to the coordinating pharmacist. The coordinating pharmacist prepared the cases for the triage meeting with the physician and home care nurse to decide on further actions. Each patient’s physician made the final decisions on medication changes needed.

Outcomes were measured as changes in medication risks: use of potentially inappropriate medications and psychotropics; anticholinergic and serotonergic load; drug-drug interactions.

**Results:**

Participants (*n* = 129) characteristics: mean age 82.8 years, female 69.8%, mean number of prescription medicines in use 13.1. The intervention did not show an impact on the medication risks between the original intervention group and the control group in the intention to treat analysis, but the per protocol analysis indicated tendency for effectiveness, particularly in optimizing central nervous system medication use. Half (50.0%) of the participants with a potential need for medication changes, agreed on in the triage meeting, had none of the medication changes actually implemented.

**Conclusion:**

The care coordination intervention used in this study indicated tendency for effectiveness when implemented as planned. Even though the outcome of the intervention was not optimal, the value of this paper is in discussing the real world experiences and challenges of implementing new practices in home care.

**Trial registration:**

ClinicalTrials.gov (NCT02545257). Registered September 9 2015.

## Background

Home care services for older adults are a critically important part of healthcare [1, 2]. In Finland, home care services are mostly based on regular, even five-time daily visits by home care practical nurses (PNs), coordinated by home care nurses. The allocation of physicians’ time for clients is limited. As populations are aging, a growing number of home care clients are frail and use multiple, complex medications [1, 3]. Combined with the lack of coordination of care this may pose uncontrolled polypharmacy and potential patient safety risks [4–6]. E.g., in Finland, the lack of coordination was identified as the major challenge in the National Medicines Agency’s program to optimize medicine use among older adults [7]. The system-based factors were found to lead to a situation where no one in the care team can concentrate on an individual patient’s medications [4, 7]. These challenges have been addressed in the current Government Program based Rational Pharmacotherapy Action Plan by 2022 with improved coordination of care as its primary goal [2, 8]. This requires new ways to organize the care of older adults, e.g. in home care context.

Despite the challenges in the coordination of medication management processes in primary care, little research has focused on prospective medication risk management of older home care clients in this setting. Coordination of care of home-dwelling older adults has been studied from a nursing approach with various interventions focusing on disease management, transitional care and self-management education programs [9, 10], but a prospective medication risk management approach has been out of their scope. Another widely researched topic has been collaborative medication review interventions. Even though several recent systematic reviews and meta-analyses have summarized evidence on the effectiveness of collaborative medication reviews in various healthcare settings [7, 11–13], studies lack descriptions of the procedures [7]. Furthermore, medication reviews are shown to be often operationalized as isolated cross-sectional assessments of patients’ medications without proper integration and coordination with other patient care procedures, which has minimized their effectiveness [14]. The aim of this randomized controlled trial (RCT) was to assess the impact of enhanced coordination of care on the outcomes of prospective medication risk management of older home care clients in primary care. The hypothesis was that a care coordination intervention would reduce the number of medication risks identified in drug regimens of older adults over a one-year period.

## Methods

### Design and context

This study was a clustered RCT with a one-year follow-up period. System-based risk management theory and action research method were applied to construct the prospective coordinated medication risk management (CoMM) procedure used in this RCT [6, 15, 16]. The developed procedure coordinates existing resources in medication risk management of older home care clients. One of the goals was to enhance community pharmacists’ collaboration with home care and enhance their contribution to prospective medication risk management [6].

This demonstration project was carried out in the municipality of Lohja, Finland (48,000 inhabitants and 384 clients receiving regular home care in 2015). The Lohja Home Care unit presents a typical home care system within publicly funded healthcare in Finland covering the entire population [17]. The home care unit is divided into five service areas, each having a leading nurse, nurses and practical nurses (PNs) who mostly conduct home visits [18, 19]. Lohja is a homogenous municipality, without major regional socioeconomic differences. The home care service areas are basically equivalent in characteristics of the clients and standard care provided. Other units involved in this study are the local health center and a community pharmacy.

### Participants and randomization

Participants were recruited by home care nurses and PNs between September 2015 and December 2015 [6]. The recruited participants fulfilled the following inclusion criteria: > 65 years old; receives regular home care; uses at least one prescription medicine; and, a written informed, voluntary consent to participate, given by the participant or his/her closest proxy [6].

**Fig. 1:**
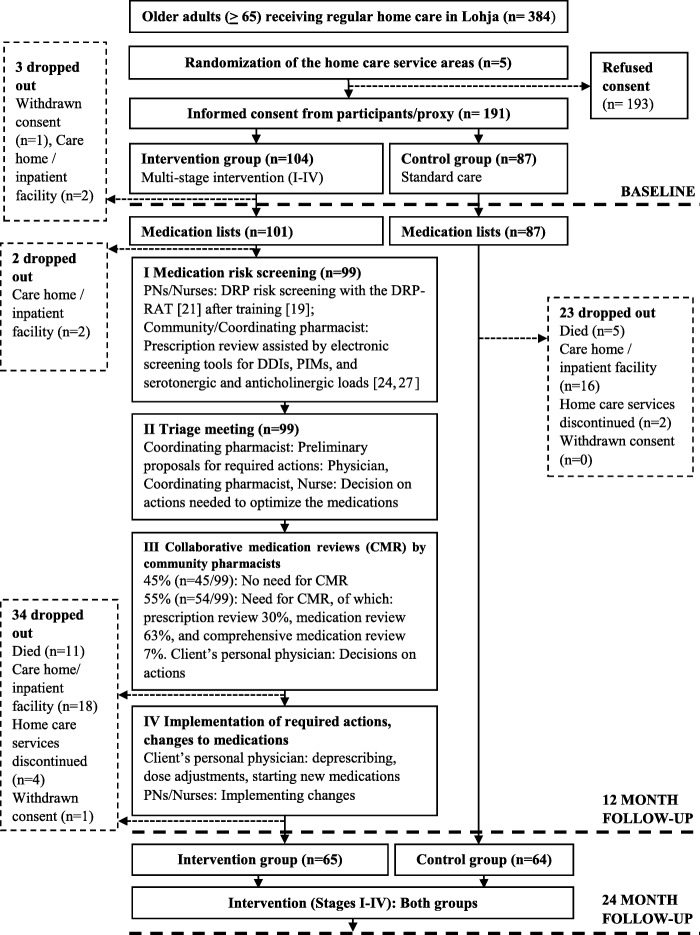
Study flow chart. PN = Practical Nurse, DRP-RAT = Drug-related problem risk assessment tool, DDI = Drug-drug interaction, CMR = Collaborative Medication review

Participants were cluster randomized to intervention and control groups by home care service area (Fig. 1) [6]. The study was considered as open-label. The intervention group (IG) received the intervention (CoMM) [6] during the first year, while the control group (CG) received standard home care. After the 12-month follow-up, the CG received the same intervention.

### Standard home care

Standard home care consists of care provided by the home care units. Based on a client’s needs, it contains help in daily activities and medicine use. Practical nurses (PNs) mostly conduct home visits. The frequency of the visits depends on the client’s needs, varying from weekly visits to five-times a day visits. Allocation of physicians’ time to home care patients is limited and nurses typically consult physicians at the health center as needed to make care decisions, also concerning pharmacotherapy. PNs or nurses obtain medicines for home care clients from the private community pharmacy. In most cases, medicines are dispensed as prepacked sachets by an automated dose dispensing system (ADD) [20]. Otherwise, nurses or practical nurses manually dispense the medicines to a doset during their home visits. Some clients are capable to take their medicines without assistance, but usually the nurse or PN must come to their home, retrieve the medicines from the doset or ADD-prepacked sachets [20] and give them to the client. Community pharmacies cooperate with home care units by dispensing medicines, and assisting in medication management, e.g., in renewing prescriptions, solving drug-related problems (DRPs) and training home care personnel [20].

### Intervention

In the CoMM procedure, the core was a triage that customized medication reviews according to each home care clients’ needs and enhanced use of existing resources [6]. First, PNs were trained to screen clinically significant DRPs, i.e., DRPs needing intervening actions, during their routine home visits and report their findings to the home care team consisting of a leading nurse, nurses and PNs, which forwarded the risk screenings to the coordinating pharmacist (Fig. 1) [19, 21]. In the DRP risk screening at home, PNs interviewed their clients by using the Drug Related Problem Risk Assessment Tool (DRP-RAT) [21]. The following information was gathered: (1) Basic Client Data, (2) Potential Risks for DRPs in Medication Use (symptoms potentially suggestive of ADRs; and medicines potentially harmful or problematic for adults aged > 65 years), (3) Characteristics of the Client’s Care and Adherence (e.g. involvement in one’s care, health care units recently visited, number of care-taking physicians), and (4) Recommendations for Actions to resolve DRPs (e.g. using dose dispensing device, visiting the personal physician, weekly control visits by a home care nurse) [21].

PNs also conducted medication reconciliation and compiled medication lists. Based on the PNsʼ reports and reconciled medication lists a clinically trained coordinating pharmacist, with the help of the community pharmacists, identified clients whose medications needed a physician’s consultation. Electronic medication risk management tools were utilized in the identification of clinically significant medication-related risks [6]. If critical medical concerns were identified, they were advised to be managed without delay by contacting client’s personal physician.

### Triage meetings and collaborative medication reviews

The physician’s consultations took place in collaborative triage meetings involving the physician, the coordinating pharmacist and a home care nurse. It was possible to agree on actions needed for 50–70 home care clients in each consultation meeting lasting 2 h [6]. The further actions usually included more comprehensive, customized medication reviews that were categorized by modifying Clyne et al. categorization [6, 22].

The first step was a prescription review in which community pharmacists identified potential DRPs such as PIMs, drug-drug interactions (DDIs), serotonergic and anticholinergic loads from the medication lists by using computerized tools integrated into the dispensing system at the community pharmacy. They reported on clients with clinically significant DRPs to each one’s physician in a separate brief written report, which was delivered by nurses [6].

In a more comprehensive medication review, community pharmacists used, in addition to medication lists, medical records and results of the DRP risk assessments by PNs to make recommendations to solve potential DRPs (e.g., adjust appropriate doses with renal dysfunction, adjust use of PIMs potentially inducing adverse symptoms). Nurses took the written medication review reports from the pharmacy to each client’s physician. After that, the community pharmacist involved discussed the findings and recommendations with the physician on the phone.

The most comprehensive medication review, i.e. clinical medication review (CMR), was conducted for clients with complicated medications that included many potential risks, such as inappropriate medicines for older adults according to Beers Criteria [23], medicines with inappropriate duration (usually too long duration), inappropriate combination of medicines, e.g., clinically significant DDIs [24], and symptoms potentially suggestive of clinically significant ADRs [21] that posed them to a high risk of harm. This procedure was performed by community pharmacists specially trained and accredited for conducting CMRs [25]. In addition to above-mentioned patient data, they used more extensively medical records, such as diagnoses, laboratory results, and medication history for reviewing the medication. They also made a home visit and interviewed the client to obtain a comprehensive medication profile [6, 22]. After conducting the review, the community pharmacist prepared a structured, written case report with recommendations for the physician, and discussed it jointly in a face-to-face case meeting to decide on actions which were documented with a follow-up plan.

After the collaborative medication reviews, each client’s physician made the final decisions on the changes to the medication regimens. In some cases, the physician wanted to meet the home care client and discuss the changes. In most cases, home care nurses who knew the clients discussed the changes with them and implemented the changes according to the physician’s orders. This was a normal routine in the home care context In Finland, due the limited physician resources. The more detailed description of the intervention can be found in the study protocol paper [6].

### Clients’ preferences in the intervention

In the risk assessment stage, PNs interviewed the clients using the DRP-RAT tool, which considers client’s view, particularly concerns of potential drug-induced adverse symptoms and poor adherence [21]. Thus, clients’ views were considered in cases where patient’s condition allowed their interview, although physicians made the final clinical decisions. Some clients with severe cognition impairment were not able to participate in the decision making related to their medication regimen. In those cases, decisions were jointly made by the physician, the nurse and the community pharmacist involved in the care of the client.

### Outcome measures

This study focused on clinically significant medication-related risks (i.e., DRPs needing intervening actions) as primary outcomes for assessing the effectiveness of the intervention. Aspects assessed revolved around potentially inappropriate medications (PIMs), excessive use of psychotropics, anticholinergic and serotonergic load and clinically significant drug-drug interactions (DDIs) (Table 1).

**Table 1:** Clinically significant medication-related risks that were assessed as outcomes in this study

Outcome	Aspects assessed
Number of all medications	The total number of regular and pro re nata (when required) medications that have been prescribed (i.e. prescribed medication that is scheduled), over-the-counter and herbal products are not included
Use of harmful medications	Included Beers Criteria medications [23], psychotropic medications and anticholinergic medications according to Puustinen et al. 2012 [26].
Use of Beers Criteria [23] medications	Potentially inappropriate medicines for older adults according to Beers Criteria [23].
Use of CNS medications	Opioids (ATC code N01AH, N02A, N02BE51, R05DA, R05FA); anticholinergic drugs according to Puustinen et al. 2012 [26], antiepileptics (ATC code N03A); BZDs and related drugs (ATC codes N05BA, N05CD, N03AE01, N05CF, A03CA, C01DA70, M05AA51, N06CA01, N02BA71); antidepressants (ATC codes N06A, N06CA); antipsychotics (ATC codes N05A, N06CA01).
Use of Psychotropic medications Proportion of study participants using > 3 psychotropic medications, n (%)	BZDs and related drugs (ATC codes N05BA, N05CD, N03AE01, N05CF, A03CA, C01DA70, M05AA51, N06CA01, N02BA71); antidepressants (ATC codes N06A, N06CA); antipsychotics (ATC codes N05A, N06CA01) [26]
Proportion of study participants using > 2 serotonergic medications, n (%)	Serotonergic medications according to Salko database [27].
Proportion of study participants using anticholinergic medications, n (%)	Anticholinergic medicines according to Puustinen et al. 2012 [26].
Proportion of study participants using antipsychotics, n (%)	ATC codes N05A, N06CA01
BZD users, n (%)	BZDs and related drugs (ATC codes N05BA, N05CD, N03AE01, N05CF, A03CA, C01DA70, M05AA51, N06CA01, N02BA71) [26]
Opioid users, n (%)	ATC codes N01AH, N02A, N02BE51, R05DA, R05FA
PPI user, n (%)	ATC A02BC
Prevalence of clinically significant drug-drug interactions (SFINX class D) [24]	Interactions that should be avoided or used with caution [24].

*CNS* central nervous system, *ATC* The Anatomical Therapeutic Chemical classification system, WHO 2018 [28], *BZD* benzodiazepine, *PPI* proton-pump inhibitor

All clinical measures used in this study were administered by the PNs and nurses during a separate home visit (Table 2). A majority of the applied outcome measures were in normal clinical use in Lohja Home Care as indicated in Table 2.

**Table 2:** Baseline Characteristics of Participants (including all participants assessed at baseline and at 12-month follow-up)

	N	Total	Intervention Group (*N* = 65)	Control Group (*N* = 64)	*p*^*c*^
Female, n (%)	129	90 (69.8)	44 (67.7)	46 (71.9)	0.61
Mean age; years, (SD), range	129	82.8 (7.1), 65–96	81.6 (7.1), 65–95	84.0 (6.8), 67–96	0.05
Age group, n (%)	129				0.08
65–69		8 (6.2)	5 (7.7)	3 (4.7)	
70–74		9 (7.0)	5 (7.7)	4 (6.3)	
75–79		17 (13.2)	13 (20.0)	4 (6.3)	
80–84		41 (31.8)	21 (32.3)	20 (31.3)	
85-		54 (41.9)	21 (32.3)	33 (51.6)	
Living alone, n (%)	126	104 (80.6)	53 (84.1)	51 (81.0)	0.64
Rava index^a,b^ [29], mean (SD)	128	1.98 (0.61)	2.19 (0.63)	1.77 (0.51)	< 0.001
MD		1.83	2.08	1.59	
MNA Screening^b^ [30]	127				0.14
Normal nutritional status (12–14 points), n (%)		65 (51.2)	30 (46.9)	35 (55.6)	
At risk of malnutrition (8–11 points), n (%)		54 (42.5)	32 (50.0)	22 (34.9)	
Malnourished (0–7 points), n (%)		8 (6.3)	2 (3.1)	6 (9.5)	
MMSE^b^ [31]	126				0.44
25–30 (no cognitive impairment), n (%)		44 (34.9)	19 (30.7)	25 (39.1)	
18–24 (mild cognitive impairment), n (%)		70 (55.6)	38 (61.3)	32 (50.0)	
0–17 (severe cognitive impairment), n (%)		12 (9.5)	5 (8.1)	7 (10.9)	
GDS-15^b^ [32] > 6 (suggestive of depression), n (%)	127	39 (30.7)	21 (33.3)	18 (28.1)	0.52
Proportion with orthostatic hypotension [33], n (%)	106	24 (22.6)	14 (29.2)	10 (17.2)	0.14
UDI-6 [34], mean (SD)	126	3.2 (4.0)	3.3 (4.4)	3.2 (3.6)	0.92
MD		2.0	2.0	2.0	
AUDIT-C [35], mean (SD)	128	0.9 (1.7)	1.2 (1.9)	0.7 (1.5)	0.59
MD		0.0	0.0	0.0	
The five-times-sit-to-stand test^b^ [36, 37]. Participants with inability to complete the test, n (%)	128	52 (40.6)	32 (50.0)	20 (31.3)	0.03
Medication use	129				
Number of regular medications, mean (SD)		10.1 (3.3)	10.4 (3.3)	9.8 (3.2)	0.25
Number of pro re nata medications, mean (SD)		3.0 (1.8)	3.1 (1.7)	2.9 (1.8)	0.54
Number of all medications, mean (SD)		13.1 (4.1)	13.5 (3.9)	12.7 (4.3)	0.25
Number of all medication, classified					0.53
1–6, n (%)		5 (3.9)	1 (1.5)	4 (6.3)	
7–9, n (%)		16 (12.4)	7 (10.8)	9 (14.1)	
10–15, n (%)		76 (58.9)	40 (61.5)	36 (56.3)	
16+, n (%)		32 (24.8)	17 (26.2)	15 (23.4)	

*SD* standard deviation, *MD* median, *MNA* The Mini Nutritional Assessment, *MMSE* Mini Mental State Examination, *GDS-15* Geriatric Depression Scale, *UDI-6* (Urinary Distress Inventory), *AUDIT-C* (Alcohol Use Disorder Identification Test, version C)

^a^Rava index describes the need of help based on functional ability (scale 1.29–4.03; results 1.50–1.99 mean need for regular help); ^b^Measure used in usual clinical practice in Lohja Home Care; ^c^Differences between the groups were tested with Chi-squared test or Fischer exact test in categorical variables and with Mann-Whitney test or two-sample t-test in continuous variables

### Statistical analyses

Up-to date medication lists gathered from both intervention and standard care group participants at baseline and at 12-month follow-up point were analyzed for medication-related risks. All participants who were assessed at baseline and at 12-month follow-up were included in the analyses. Since medication changes proposed in the medication reviews were only partly implemented, data was analyzed with the intention to treat (ITT) and per protocol analysis.

#### 1 Baseline analyses

Baseline analyses were conducted to compare the participants’ characteristics and the clinically significant medication risks between the IG and CG participants. Participants’ characteristics included demographics and the following clinical outcomes: functional ability (Rava) [29]; physical performance (The five-times-sit-to-stand test) [36, 37]; Mini Mental State Examination (MMSE) [31]; Geriatric Depression Scale − 15 (GDS-15) [32]; The Mini Nutritional Assessment (MNA) [30]; Urinary Distress Inventory (UDI-6) [34]; Orthostatic hypotension (Short test) [33]; and Alcohol Use Disorder Identification Test, version C (AUDIT-C) [35].

Analyses were performed using a two-sample t-test for normally distributed variables and by Mann-Whitney U-test for non-normally distributed variables. Chi-square or Fisher’s exact test was used for categorical variables.

#### 2 Intention to treat- and per protocol analyses

For the ITT analysis all the participants were included in the group in which they were randomly assigned (IG or CG) regardless of whether medication changes agreed on were implemented. Per protocol analysis included only those IG participants that had at least one of the medication changes actually implemented.

Descriptive statistics (mean, median or percentages as appropriate) were used to present the participant characteristics. The changes within and between groups in continuous variables were analyzed with repeated measures analysis of variance. Dichotomous outcomes were analyzed by binary logistic regression using generalized estimating equations to account for the correlation between the repeated measurements. Results are expressed using odds ratios (OR) with 95% confidence intervals (CI). ITT analyses were adjusted for functional ability and the use of antiepileptic medications, and per protocol analyses were adjusted for functional ability, use of central nervous system medications (CNS-medications), GDS-15 and MNA due to group differences at the baseline. In the longitudinal analysis were included participants with baseline measurement and at least one follow-up measurement at 12-month follow-up point. Two-sided statistical tests with a 5% level of significance were used.

## Results

### Study participants and attrition rate

Of 384 eligible home care clients, 191 (49.7%) clients or their proxies provided written consent to participate (Fig. 1). The IG included 104 participants, of which three dropped out before baseline data gathering. The CG included 87 study participants. There was a remarkable attrition rate, with 59 participants (31.4%) lost to follow-up at 12 months. In the IG, attrition rate was 35.6% (*n* = 36) and in the CG 26.4% (*n* = 23) (*p* = 0.18) (Fig. 1). Drop-out analysis between the IG and CG participants did not show statistically significant differences in baseline characteristics (data not shown). Number of participants with baseline and 12-months follow-up data available, included into the analyses, was 129.

At baseline, the characteristics of the participants (*n* = 129) in the IG and CG did not differ, despite differences in functional ability (Rava) (*p* = 0.01) and the use of antiepileptic medications (ATC code N03A) (*p* = 0.003), which were adjusted in the ITT analysis. The mean age of the participants (n = 129) was 82.8 years (SD 7.05), 82.5% (*n* = 104), were living alone and 69.8% (*n* = 90) were women (Table 2). The mean number of prescription medications (regular and pro re nata, i.e. when required) was 13.5 (SD 3.87) in the IG compared with 12.7 (SD 4.30) in the CG (*p* = 0.25).

The baseline characteristics of the IG (*n* = 27) and CG participants (*n* = 64), included in the per protocol analysis, differed in functional ability (Rava) (*p* = 0.006), use of CNS-medication (*p* = 0.02), GDS-15 (*p* = 0.03), and MNA (*p* = 0.005), which were adjusted.

### Use of PIMs at baseline

Use of PIMs, excessive use of psychotropics, and high anticholinergic and serotonergic load was common in both groups at baseline (Table 3). Prevalence of Beers Criteria [23] medication use was 93.9% in IG and 90.6% in CG (*p* = 0.53*)*; anticholinergic use was 27.7% in IG and 18.8% in CG (*p* = 0.23); > 3 psychotropics use was 20.0% in IG and 9.4% in CG (*p* = 0.09) (Table 3). The most commonly used Beers Criteria medications were proton pump inhibitors (PPIs), when used for longer than 2 months without precise indication: this was the case for 50.4% (*n* = 60) of the Beers Criteria medication users (*n* = 119).

**Table 3:** Outcomes describing potentially inappropriate medicine use at baseline and after 12 months in the intervention and control groups and significance of changes within and between the groups during the follow-up

Medication in use	Intervention group (*N* = 65)			Control group (*N* = 64)					
	Baseline	At 12 months	Adjusted mean change [95% CI] or adjusted OR (95% CI) for change	p for change	Baseline	At 12 months	Adjusted mean change [95% CI] or adjusted OR (95% CI) for change	p for change	P^h^ for the difference in change between the groups
Number of all medications, mean (SD)	13.5 (3.9)	14.1 (3.8)	0.77 [0.05–1.48]	0.04	12.7 (4.30)	12.95 (4.03)	0.52 [−0.37–1.41]	0.25	0.59
Harmful medication^a^ user, n (%)	56 (86.2)	57 (87.7)	1.15 (0.63–2.10)	0.66	51 (79.7)	51 (79.7)	1.00 (0.63–1.60)	1.00	0.73
Beers Criteria [23] medication user, n (%)	61 (93.9)	63 (96.9)	2.12 (0.34–13.2)	0.42	58 (90.6)	57 (89.1)	0.84 (0.39–1.82)	0.66	0.36
Psychotropic medication^b^ user, n (%)	43 (66.2)	45 (69.2)	1.15 (0.78–1.72)	0.48	32 (50.0)	30 (46.9)	0.88 (0.62–1.25)	0.48	0.32
Anticholinergic medication^c^ user, n (%)	18 (27.7)	17 (26.2)	0.92 (0.58–1.42)	0.74	12 (18.8)	12 (18.8)	1.00 (0.75–1.33)	1.00	0.77
CNS medication^d^ user, n (%)	56 (86.2)	53 (81.5)	0.69 (0.34–1.41)	0.31	45 (70.3)	43 (67.2)	0.86 (0.49–1.50)	0.59	0.64
Therapeutic duplication									
Proportion using > 3 psychotropic medications^b^, n (%)	13 (20.0)	12 (18.5)	0.81 (0.53–1.22)	0.31	6 (9.4)	5 (7.8)	0.82 (0.41–1.62)	0.57	0.97
Proportion using > 2 serotonergic medications [27], n (%)	13 (20.0)	16 (24.6)	1.35 (0.88–2.06)	0.17	5 (7.8)	7 (10.9)	1.47 (0.69–3.16)	0.32	0.84
Use of special ATC classes									
Antipsychotics users^e^, n (%)	13 (20.0)	15 (23.1)	1.20 (0.72–2.01)	0.48	7 (10.9)	7 (10.9)	1.00 (0.64–1.57)	1.00	0.59
BZD^f^ users, n (%)	32 (49.2)	30 (46.2)	0.88 (0.62–1.25)	0.48	21 (32.8)	20 (31.3)	0.93 (0.64–1.35)	0.70	0.83
Opioid^g^ users, n (%)	18 (27.7)	17 (26.2)	0.92 (0.55–1.55)	0.76	22 (34.4)	20 (31.3)	0.87 (0.58–1.29)	0.48	0.85
PPI (ATC A02BC) user, n (%)	30 (46.2)	30 (46.2)	1.00 (0.78–1.29)	1.00	30 (46.9)	33 (51.6)	1.21 (0.92–1.59)	0.17	0.31
Drug-drug interactions (DDI)									
Clinically significant DDI (class D) [24], n (%)	7 (10.8)	6 (9.2)	0.84 (0.39–1.79)	0.65	1 (1.6)	0 (0)	NA	0.32^i^	NA

*SD* standard deviation, *OR* Odds ratio, *CI* Confidence interval, *CNS* central nervous system, *BZD* benzodiazepine, *PPI* proton-pump inhibitor medicine

^a.^Harmful medications Included 1) Beers Criteria medications [23], 2) ^b.^psychotropic medications [26] (Included ^f^*BZDs and related drugs*: ATC codes N05BA, N05CD, N03AE01, N05CF, A03CA, C01DA70, M05AA51, N06CA01, N02BA71; *Antidepressants*: ATC codes N06A, N06CA; ^e^*Antipsychotics*: ATC codes N05A, N06CA01); 3) ^c^ Anticholinergic medications [26]: ATC codes: N04A, N05AA01, N05AA02, N05AB01, N05AB02, N05AB03, N05AB04, N05 AC01, N05 AC02, N05AF01, N05AF03, N05AF05, N05BB01, N06AA04, N06AA06, N06AA09, N06AA12, N02AG, A03AA, A03AB, A03AX03, A03B, A03CA, A03CB31, A03DA, A03FA01, A04AD01, A04AD12, C01BA01, C01BA03, C01BA51, C01BA71, R03BB, M03B, G04BD, S01FA, R01BA01, R01BA51, R06AB01, R06AE03, R06AE53). If the medication appeared in two or more criteria, they were considered only once

^d^CNS medications: ^g^Opioids (ATC codes N01AH, N02A, N02BE51, R05DA, R05FA), ^c^anticholinergics [26]; antiepileptics (ATC N03A); ^f^BZDs and related drugs (ATC codes N05BA, N05CD, N03AE01, N05CF, A03CA, C01DA70, M05AA51, N06CA01, N02BA71); Antidepressants (ATC codes N06A, N06CA); ^e^Antipsychotics (ATC codes N05A, N06CA01)

^h^Adjusted for functional ability Rava and the use of antiepileptic medications (ATC code N03A).^i^ McNemar test due to zero frequencies

### Effect of the intervention (CoMM) on the use of PIMs (ITT analyses)

No clinically significant medication-related risks needing collaborative medication review were found for 45.5% (*n* = 45) of the 99 IG participants who had DRP-RAT assessment available (Fig. 1). Of the remaining 54.5% (*n* = 54), prescription review was needed in 29.6% (*n* = 16), medication review in 63.0% (*n* = 34), and comprehensive medication review in 7.4% (n = 4) of the cases.

Mean number of all medications in use increased in both groups over the 12-month follow-up period: in IG from 13.5 to 14.1 (adjusted mean change 0.77 95% CI 0.05–1.48; *p* = 0.04) and in CG from 12.7 to 13.0 (adjusted mean change 0.52 95% CI -0.37-1.41; *p* = 0.25) (Table 3). The prevalence of PIM use remained mainly constant in both groups. No significant changes (*p* < 0.05) were found in any selected medication-related outcomes between the IG and CG in the ITT analyses (Table 3).

### Per protocol analyses

Per protocol analysis compared IG participants with at least one implemented medication change (*n* = 27) with CG participants (*n* = 64) (Table 4). No significant differences (p < 0.05) were found in medication-related outcomes between the IG per protocol (IG_pp_) and CG over the 12-month follow-up period (Table 4). However, a tendency for a decrease was found in the use of central nervous system (CNS) medications between the groups (*p* = 0.08): in IG_pp_ a decrease of 18.5 percentage points was observed (adjusted OR 0.15 95% CI 0.03–0.80), compared to a decrease of 3.1 percentage points (adjusted OR 0.81 95% CI 0.37–1.77) in CG.

**Table 4:** Outcomes describing potentially inappropriate medicine use at baseline and after 12 months in the *per protocol* intervention group and control groups and significance of changes within and between the groups during the follow-up

Medication in use	Intervention group (*N* = 27)			Control group (*N* = 64)					
	Baseline	At 12 months	Adjusted mean change [95% CI] or adjusted OR (95% CI) for change	p for change	Baseline	At 12 months	Adjusted mean change [95% CI] or adjusted OR (95% CI) for change	p for change	P^i^ for the difference in change between the groups
Number of all medications, mean (SD)	14.0 (3.9)	13.3 (3.3)	−0.02 [−1.24–1.20]	0.97	12.7 (4.3)	13.0 (4.0)	0.38 [−0.59–1.36]	0.44	0.46
Harmful medication^a^ user, n (%)	23 (85.2)	22 (81.5)	0.61 (0.13–2.88)	0.54	51 (79.7)	51 (79.7)	1.00 (0.50–2.02)	1.00	0.58
Beers Criteria [23] medication user, n (%)	26 (96.3)	25 (92.6)	0.42 (0.02–7.52)	0.56	58 (90.6)	57 (89.1)	0.82 (0.34–1.95)	0.65	0.67
Psychotropic medication^b^ user, n (%)	21 (77.8)	19 (70.4)	0.47 (0.04–5.24)	0.54	32 (50.0)	30 (46.9)	0.56 (0.11–2.72)	0.47	0.90
Anticholinergic medication^c^ user, n (%)	8 (29.6)	5 (18.5)	0.62 (0.25–1.56)	0.31	12 (18.8)	12 (18.8)	1.00 (0.72–1.39)	1.00	0.34
CNS medication^d^ user, n (%)	25 (92.6)	20 (74.1)	0.15 (0.03–0.80)	0.03	45 (70.3)	43 (67.2)	0.81 (0.37–1.77)	0.59	0.08
Therapeutic duplication									
Proportion using > 3 psychotropic medications^b^, n (%)	5 (18.5)	2 (7.4)	0.35 (0.11–1.10)	0.07	6 (9.4)	5 (7.8)	0.82 (0.42–1.62)	0.56	0.21
Proportion using > 2 serotonergic medications [27], n (%)	6 (22.2)	7 (26.0)	1.28 (0.56–2.92)	0.56	5 (7.8)	7 (10.9)	1.49 (0.68–3.26)	0.32	0.79
Use of special ATC classes									
Antipsychotics users^e^, n (%)	6 (22.2)	4 (14.8)	0.59 (0.20–1.69)	0.32	7 (10.9)	7 (10.9)	1.00 (0.61–1.65)	1.00	0.37
BZD^f^ users, n (%)	15 (55.6)	10 (37.0)	0.43 (0.21–0.91)	0.03	21 (32.8)	20 (31.3)	0.89 (0.47–1.67)	0.71	0.15
Opioid^g^ users, n (%)	11 (40.7)	7 (26.0)	0.49 (0.21–1.11)	0.09	22 (34.4)	20 (31.3)	0.86 (0.57–1.30)	0.47	0.23
PPI (ATC A02BC) user, n (%)	13 (48.2)	13 (48.2)	1.00 (0.62–1.61)	1.00	30 (46.9)	33 (51.6)	1.23 (0.91–1.66)	0.18	0.47
Drug-drug interactions (DDI**)**									
Clinically significant DDI (class D) [24], n (%)	2 (7.4)	2 (7.4)	1.00 (0.21–4.67)	1.00	1 (1.6)	0 (0.0)	NA	0.32^h^	NA

*SD* standard deviation, *OR* Odds ratio, *CI* Confidence interval, *CNS* central nervous system, *BZD* benzodiazepine, *PPI* proton-pump inhibitor, *NA* not available

^a^ Harmful medications Included 1) Beers Criteria medications [23], 2) ^b^ psychotropic medications [26] (Included ^f^*BZDs and related drugs*: ATC codes N05BA, N05CD, N03AE01, N05CF, A03CA, C01DA70, M05AA51, N06CA01, N02BA71; *Antidepressants*: ATC codes N06A, N06CA; ^e^*Antipsychotics*: ATC codes N05A, N06CA01); 3) ^c^ Anticholinergic medications [26]: ATC codes: N04A, N05AA01, N05AA02, N05AB01, N05AB02, N05AB03, N05AB04, N05 AC01, N05 AC02, N05AF01, N05AF03, N05AF05, N05BB01, N06AA04, N06AA06, N06AA09, N06AA12, N02AG, A03AA, A03AB, A03AX03, A03B, A03CA, A03CB31, A03DA, A03FA01, A04AD01, A04AD12, C01BA01, C01BA03, C01BA51, C01BA71, R03BB, M03B, G04BD, S01FA, R01BA01, R01BA51, R06AB01, R06AE03, R06AE53). If the medication appeared in two or more criteria, they were considered only once

^**d**^ CNS medications: ^g^Opioids (ATC codes N01AH, N02A, N02BE51, R05DA, R05FA), ^c^anticholinergics [26]; antiepileptics (ATC N03A); ^f^BZDs and related drugs (ATC codes N05BA, N05CD, N03AE01, N05CF, A03CA, C01DA70, M05AA51, N06CA01, N02BA71); Antidepressants (ATC codes N06A, N06CA); ^e^Antipsychotics (ATC codes N05A, N06CA01). ^h^McNemar test due to zero frequencies. ^i^Adjusted for functional ability Rava, CNS medications, GDS-15, MNA

### Analyses within the per protocol group (n = 27)

In the analyses within the IG_pp_, in addition to a decrease in CNS use (from 92.6 to 74.1%; adjusted OR 0.15, 95% CI 0.03–0.80; *p* = 0.03), the use of benzodiazepines (BZDs) decreased from 55.6 to 37.0% (adjusted OR 0.43, 95% CI 0.21–0.91; p = 0.03). A tendency for a decrease within the IG_pp_ (*p* < 0.10) was shown in the following outcomes: proportion of persons using > 3 psychotropic medications decreased from 18.5 to 7.4% (*p* = 0.07); and opioid use decreased from 40.7 to 26.0% (*p* = 0.09) (Table 4).

## Discussion

The intervention did not show an impact on the use of PIMs between the original intervention group and the control group in the intention to treat analysis, but the per protocol analysis indicated tendency for effectiveness, particularly in optimizing CNS medication (especially in BZD) use during a 12-month follow-up. As the original IG included many home care clients whose medication changes were not actually implemented as proposed (50% of the intervention group participants), the intervention was incomplete for them. Thus, per protocol analysis is a better predictor of the effectiveness of the coordinated home care model than a comparison between the original intervention group and the control group.

### High prevalence of PIM use

Our baseline findings demonstrate a high prevalence of PIM use. Particularly, the prevalence of potentially inappropriate psychotropic medication use was high (58.1%, *n* = 75) in the entire study population (IG and CG, *n* = 129) included in the intention to treat analysis. Most common was potentially inappropriate BZD use (41.1%) and antidepressant use (36.4%). National register-based data shows that long-term use of BZDs is the major PIM-related concern in Finland, particularly the use of temazepam [38]. A more recent study indicates a declining trend in the long-term BZD use over the last years (2006–2014), but the long-term BZD use has remained constant among the older adults [39]. The decline has not been uniform between the substances: the long-term use of clonazepam and zolpidem has even increased [39]. These findings indicate an urgent need for effective deprescribing interventions that should be actively promoted to make them part of the routine clinical practice. There are recent promising results of successfully reducing long-term BZD use in older adults by community-based interventions in primary care [40, 41].

Inappropriate use of antipsychotics (APs) is another major concern in geriatric pharmacotherapy which can also be seen in our data (baseline users: 15.5%, *n* = 20). APs are usually prescribed for behavioral disorders with dementia but the use may even continue for years without proper follow-ups [42]. International criteria, e.g. Beers Criteria, have updated their recommendations on the use of APs in recent years: use should be avoided unless nonpharmacological options have failed or are not possible and the person is threatening substantial harm to self or others [23]. However, Finnish guidelines are not as strict concerning AP use in older adults as the most recent international guidelines [43]. This may partly explain their wide use among older outpatients and inpatients in Finland [42]. It would be important to reconsider our domestic guidelines and care practices to meet current international standards in AP use.

Another contributing factor to excessive use of antipsychotics in older adults is culture of care [44, 45]. Our experience in Lohja Home Care was that some of the physicians and nurses were reluctant to actually stop the AP treatment even though the potential need for deprescribing was agreed on in the triage meeting. As previous studies have shown, this may be due to concern about stopping medications started and prescribed by other physicians, limited knowledge about how to stop APs, and concern about a relapse of behavioral disorders [45–47]. Further research is needed to better understand these systems-based factors influencing AP use that can lead to unnecessary and harmful long-term medications.

### Triage

The core of the intervention in this study was triage meetings that proved to be a feasible method for customizing comprehensiveness of collaborative medication reviews (CMRs) for older home care clients according to their clinical needs while minimizing physician’s time required [6]. Of the older home care clients, 45.5% had no need for more comprehensive medication reviews. The triage enabled focusing on clients with clinically significant DRPs instead of comprehensively reviewing medications of all clients, as has been the case in many previous studies [7].

### Implementation of the intervention

We experienced challenges in implementing the new procedure. This was also seen in the analysis of the effectiveness of the intervention. Physicians’ limited resources, partly reluctant attitudes and weak engagement to the new, more collaborative medication management practice were evaluated as the main contributing factors for intervention not being implemented. Some physicians were reluctant and did not approve and implement any of the recommended clinically significant medication changes. Thus, these factors affecting medicines optimization need further investigation. This trial represents real world and has features of pragmatic trials, which frequently include complex interventions, involving the skills and experience of various health care providers to deliver the intervention [48]. Our experience is that implementation of this kind of new coordinated procedure requires long-term and goal-oriented commitment of all healthcare providers involved to break organizational barriers and change working behaviors and patterns. Educational needs in both geriatric pharmacotherapy and understanding system-based medication risk management were identified in all participating health care providers and community pharmacists [6]. The most striking competence gap observed relates to deprescribing. Thus, a better deprescribing protocol needs to be used in future studies.

### Strength and limitations of the methods

Our study design and randomization strategy worked well. At baseline, the characteristics of the participants in the IG and the CG were similar, despite functional ability (Rava) and use of antiepileptics, which were adjusted in the ITT-analyses. We used cluster randomization to avoid contamination related to nurses and PNs. Contamination related to community pharmacists and physicians was not considered, since these professionals did not have regular encounters with the home care clients. Clustering by service area was not accounted in the data analysis.

We selected such outcome measures and follow-up periods that have been proven appropriate in previous studies [7, 14, 49]. A 12-month follow up period has shown to be long enough to implement medication changes, demonstrate potential changes in study participants’ health outcomes and sustainability of changes made in their medications. Selected measures were congruent with recent studies proposing core outcome measures for trials aiming to improve appropriate medication use in older adults [50, 51]. Our goal in the future study is to investigate whether there is an association between the intermediate measures used in this study (medication risks) and improved health/function/cognition outcomes.

A limitation of this study is a relatively small sample size, which may have affected the weak effectiveness of the intervention. Half of the eligible residents did not provide written consent to participate. High workload of the recruiting nursing staff, as well as frail and multi-morbid home care clients were evaluated as main contributing factors. It was also contributed by the high attrition rate during the first study year, due to high age and multiple morbidities of the participants. In future studies with multi-morbid and frail older adults, these methodological issues crucially influencing the power of the study needs to be considered better. The same has been observed in other studies with multi-morbid older adults [52].

We included in the analysis only participants with baseline and 12-month follow-up data available [53]. Poor implementation of recommended medication changes was the rationale for conducting per protocol analyses, including only participants with at least one clinically significant medication change actually implemented. As we were able to show a tendency for effectiveness in the per protocol analysis, it would be important to repeat the intervention with larger study populations to confirm the findings.

This demonstration study was carried out by involving in the intervention only one community pharmacy operating in Lohja. This strategy was chosen to keep the study design simple as adding more community pharmacies to the study would have increased risk of bias. It would be important to repeat the intervention in the home care of other municipalities and involve other community pharmacies in future research.

### Practical implications

This was a demonstration study showing preliminary and promising positive results. The procedure can be transferred to other home care units and adopted to their local circumstances. The procedure could be particularly designed to reduce CNS use in older adults as it is among the major problems in geriatric pharmacotherapy in Finland [38, 39, 42]. Further studies are needed on care culture and other contributing factors to high prevalence of PIM use and other risks for clinically significant DRPs identified in this study. Particularly, physicians’ reluctance to implement recommended medication changes in the cases with inappropriate polypharmacy, and relationship between inappropriate medication use and medication errors need further investigation.

## Conclusion

The care coordination intervention used in this study indicated tendency for effectiveness when implemented as planned, particularly in optimizing CNS medication use during a 12-month follow-up. This study demonstrates the challenges to overcome when trying to change clinical practice and improve coordination between units involved in medication management of home care clients. Even though the outcomes of the intervention were not optimal, the value of this paper is in discussing the real world experiences and challenges of implementing new practices in home care. This may help to inform future interventions and improve medication use for older persons.

## Data Availability

Not applicable. The data will not be publicly available due to regulations and agreements obtained to perform the study.

## Abbreviations

AP: Antipsychotic
ATC: Anatomical therapeutic chemical
BZD: Benzodiazepine
CG: Control group
CI: Confidence interval
CMR: Collaborative medication review
CNS: Central nervous system
CoMM-procedure: Coordinated medication risk management procedure
DDI: Drug-drug interaction
DRP: Drug-related problem
DRP-RAT: Drug-Related Problem Risk Assessment Tool
IG: Intervention group
ITT: Intention to treat
OR: Odds ratio
PIM: Potentially inappropriate medication
PIM(s): Potentially inappropriate medicine(s)
PN: Practical nurse
PP: Per protocol
PPI: Proton pump inhibitor
RCT: Randomized controlled trial
SD: Standard deviation

## References

[1] Genet N, Boerma W, Kroneman M, Hutchinson A, Saltman RB, eds. Home care across Europe: current structure and future challenges. Published 2012. Available at: http://www.euro.who.int/__data/assets/pdf_file/0008/181799/e96757.pdf?ua=1 Accessed October 22, 2019.

[2] Prime Minister’s Office Finland. Strategic Programme of Prime Minister Juha Sipilä’s Government, 29 May 2015. Government Publication 12/2015. Available at: https://valtioneuvosto.fi/documents/10184/1427398/Ratkaisujen+Suomi_EN_YHDISTETTY_netti.pdf/8d2e1a66-e24a-4073-8303-ee3127fbfcac/Ratkaisujen+Suomi_EN_YHDISTETTY_netti.pdf . Accessed October 22, 2019.

[3] Saastamoinen LK, Verho J (2015). Register-based indicators for potentially inappropriate medication in high-cost patients with excessive polypharmacy. Pharmacoepidemiol Drug Saf.

[4] World Health Organization. Towards people-centered health systems: An innovative approach for better health outcomes; 2013. Available at: http://www.euro.who.int/__data/assets/pdf_file/0006/186756/Towards-people-centred-health-systems-an-innovative-approach-for-better-health-outcomes.pdf Accessed October 22, 2019.

[5] Cadogan CA, Ryan C, Hughes CM (2016). Appropriate Polypharmacy and medicine safety: when many is not too many. Drug Saf.

[6] Toivo T, Dimitrow M, Puustinen J (2018). Coordinating resources for prospective medication risk management of older home care clients in primary care: procedure development and RCT study design for demonstrating its effectiveness. BMC Ger.

[7] Kallio SE, Kiiski A, Airaksinen MS (2018). Community Pharmacists' contribution to medication reviews for older adults: a systematic review. J Am Geriatr Soc.

[8] Ministry of Social Affairs and Health. Final report. Rational Pharmacotherapy Action Plan. Reports and memorandums of the Ministry of Social Affairs and Health 19/2018. Available at: http://stm.fi/rationaalinen-laakehoito/julkaisut . Accessed October 22, 2019.

[9] Marek KD, Stetzer F, Adams SJ, Bub LD, Schlidt A, Colorafi KJ (2014). Cost analysis of a home-based nurse care coordination program. J Am Geriatr Soc.

[10] Kim TY, Marek KD, Coenen A (2016). Identifying care coordination interventions provided to community-dwelling older adults using electronic health records. Comput Inform Nurs.

[11] Patterson SM, Hughes C, Kerse N, Cardwell CR, Bradley MC. Interventions to improve the appropriate use of polypharmacy for older people. Cochrane Database Syst Rev. 2012; 16 ;(5):CD008165. doi: 10.1002/14651858. 10.1002/14651858.CD008165.pub2 22592727

[12] Viswanathan M, Kahwati LC, Golin CE (2015). Medication therapy management interventions in outpatient settings: a systematic review and meta-analysis. JAMA Intern Med.

[13] Christensen M, Lundh A. Medication review in hospitalised patients to reduce morbidity and mortality. Cochrane Database of Systematic Reviews 2016, Issue 2. Art. No.: CD008986. DOI: 10.1002/14651858.CD008986.pub3. 10.1002/14651858.CD008986.pub3 PMC7119455 26895968

[14] Huiskes VJB, Burger DM, van den Ende CHM, van den Bemt BJF (2017). Effectiveness of medication review: a systematic review and meta-analysis of randomized controlled trials. BMC Fam Pract.

[15] Reason J (2000). Human error: models and management. Br Med J.

[16] Lewin K (1946). Action research and minority problems. J Soc Issues.

[17] Ministry of Social Affairs and Health. Health Care in Finland. Brochures of the Ministry of Social Affairs and Health, 2013. Available at: http://julkaisut.valtioneuvosto.fi/bitstream/handle/10024/69930/URN_ISBN_978-952-00-3395-8.pdf . Accessed October 22, 2019.

[18] Hammar T, Perälä ML, Rissanen P (2009). Clients’ and workers’ perceptions on clients’ functional ability and need for help: home care in municipalities. Scand J Caring Sci.

[19] Dimitrow MS, Leikola SN, Kivelä SL (2015). Feasibility of a practical nurse administered risk assessment tool for drug-related problems in home care. Scand J Public Health.

[20] Sinnemäki J, Saastamoinen LK (2014). Hannula S et al. Int J Clin Pharm.

[21] Dimitrow MS, Mykkänen SI, Leikola SNS (2014). Content validation of a tool for assessing risks for drug-related problems to be used by practical nurses caring for home-dwelling clients aged ≥65 years: a Delphi survey. Eur J Clin Pharmacol.

[22] Clyne W, Blenkinsopp A, Seal R: A Guide to Medication Review 2008. The National Prescribing Centre, the Medicines Partnership Programme 2.1. Available at: http://www.cff.org.br/userfiles/52%20-%20CLYNE%20W%20A%20guide%20to%20medication%20review%202008.pdf . Accessed October 22, 2019.

[23] The American Geriatrics Society 2015 (2015). Beers Criteria Update Expert Panel (AGS 2015). American Geriatrics Society 2015 updated beers criteria for potentially inappropriate medication use in older adults. J Am Geriatr Soc.

[24] Böttiger Y, Laine K, Andersson ML (2009). SFINX –a drug-drug interaction database designed for clinical decision support systems. Eur J Clin Pharmacol.

[25] Leikola S. Development and application of comprehensive medication review procedure to community-dwelling elderly. Doctoral Thesis, University of Helsinki, 2012. Available at: http://urn.fi/URN:ISBN:978-952-10-7698-5 . Accessed October 22, 2019.

[26] Puustinen J, Nurminen JF, Vahlberg TF (2012). CNS medications as predictors of precipitous cognitive decline in the cognitively disabled aged: a longitudinal population-based study. Dement Geriatr Cogn Dis Extra.

[27] Leikola S, Salimäki J, Teinilä T, Peura S. Salko – Medication review tool for community pharmacists. Dosis 2013; 29: 47–53. (In Finnish, Abstract in English).

[28] World Health Organisation (WHO), Collaborating Centre for Drug Statistics Methodology: The Anatomical Therapeutic Chemical (ATC) classification system. Available at: www.whocc.no. Accessed October 22, 2019.

[29] RAVA -Functional ability test for classifying the abilities of the elderly and planning necessary services. Finnish Consulting Group Available at: http://www.fcg.fi/eng/expertise/welfare_and_ict_services/classification_products. Accessed October 22, 2019.

[30] Vellas B, Guigoz Y, Garry PJ (1999). The mini nutritional assessment (MNA) and its use in grading the nutritional state of elderly patients. Nutrition.

[31] Folstein MF, Folstein SE, McHugh PR (1975). “Minimental state”. A practical method for grading the cognitive state of patients for the clinician. J Psychiatr Res.

[32] Kurlowicz L, Greenberg SA (2007). The geriatric depression scale. Am J Nurs.

[33] Freeman R, Wieling W, Axelrod FB (2011). Consensus statement on the definition of orthostatic hypotension, neurally mediated syncope and the postural tachycardia syndrome. Clin Auton Res.

[34] Uebersax JS, Wyman JF, Shumaker SA (1995). Short forms to assess life quality and symptom distress for urinary incontinence in women: the incontinence impact questionnaire and the urogenital distress inventory. Continence program for women research group. Neurourol Urodyn.

[35] Bush K, Kivlahan DR, McDonell MB (1998). The AUDIT alcohol consumption questions (AUDIT-C): an effective brief screening test for problem drinking. Arch Intern Med.

[36] Csuka M, McCarty DJ (1985). Simple method for measurement of lower extremity muscle strength. Am J Med.

[37] Guralnik JM, Simonsick EM, Ferrucci L (1994). A short physical performance battery assessing lower extremity function: association with self-reported disability and prediction of mortality and nursing home admission. J Gerontol.

[38] Leikola S, Dimitrow M, Lyles A, Pitkälä K, Airaksinen M (2011). Potentially inappropriate medication use among finnish non- institutionalized people aged =65 years: a register-based, cross-sectional, national study. Drugs Aging.

[39] Kurko TA, Saastamoinen LK, Tuulio-Henriksson A (2018). Trends in the long-term use of benzodiazepine anxiolytics and hypnotics: a national register study from 2006 to 2014. Pharmacoepidemiol Drug Saf.

[40] Puustinen J, Lahteenmaki RF, Polo-Kantola PF (2014). Effect of withdrawal from long-term use of temazepam, zopiclone or zolpidem as hypnotic agents on cognition in older adults. Eur J Clin Pharmacol.

[41] Puustinen JA, Lahteenmaki R, Nurminen J (2018). Long-term persistence of withdrawal of temazepam, zopiclone, and zolpidem in older adults: a 3-year follow-up study. BMC Geriatr.

[42] Jalava SE, Pohjanoksa-Mäntylä M, Puustinen J, Airaksinen M, Dimitrow MS. Use of Antipsychotics among Older Adults in Finland – A Systematic Review. Finn Med J. 2018;73:1743–1748. (Finnish, abstract in English).

[43] Finnish Medicines Agency Fimea. Meds75+ database. Available at: https://www.fimea.fi/web/en/databases_and_registeries/medicines_information/database_of_medication_for_the_elderly . Accessed October 22, 2019.

[44] Nurminen J, Puustinen J, Kukola M, Kivelä SL (2009). The use of chemical restraints for older long-term hospital patients: a case report from Finland. J Elder Abuse Negl.

[45] Sawan M, Jeon Y, Chen TF. Shaping the use of psychotropic medicines in nursing homes: a qualitative study on organisational culture. Soc Sci Med. 2018. 10.1016/j.socscimed.2018.02.010. 10.1016/j.socscimed.2018.02.010 29514110

[46] Reeve E, Low LF, Hilmer SN (2016). Beliefs and attitudes of older adults and carers about deprescribing of medications: a qualitative focus group study. Br J Gen Pract.

[47] Bjerre LM, Farrell B, Hogel M (2018). Deprescribing antipsychotics for behavioural and psychological symptoms of dementia and insomnia: evidence-based clinical practice guideline. Can Fam Physician.

[48] Ford I, Norrie J (2016). Pragmatic trials. N Engl J Med.

[49] Kiiski A, Kallio S, Pohjanoksa-Mäntylä M et al. Collaborative medication management models in the rationalization of the medication therapies of the aged. Systematic review. 2016 [Finnish publication, Ministry of Social Affairs and Health, publication 2016:12]. E-publication: http://urn.fi/URN:ISBN:978-952-00-3704-8. Accessed October 22, 2019.

[50] Rankin A, Cadogan CA, In Ryan C, Clyne B, Smith SM, Hughes CM (2018). Core outcome set for trials aimed at improving the appropriateness of Polypharmacy in older people in primary care. J Am Ger Soc.

[51] Beuscart JB, Knol W, Cullinan S (2018). International core outcome set for clinical trials of medication review in multi-morbid older patients with polypharmacy. BMC Med.

[52] Juola AL, Bjorkman MP, Pylkkänen S (2015). Nurse education to reduce harmful medication use in assisted living facilities: effects of a randomized controlled trial on falls and cognition. Drugs Aging.

[53] Dumville JC, Torgerson DJ, Hewitt CE (2006). Reporting attrition in randomized controlled trials. Br Med J.

